# Application of Rice Husk Biochar and Earthworm on Concentration and Speciation of Heavy Metals in Industrial Sludge Treatment

**DOI:** 10.3390/ijerph192013463

**Published:** 2022-10-18

**Authors:** Xingming Wang, Zhaoxia Chu, Tingyu Fan, Shuying Liang, Gang Li, Jiamei Zhang, Quan Zhen

**Affiliations:** 1 State Key Laboratory of Safety and Health for Metal Mines, Sinosteel Maanshan General Institute of Mining Research Company Limited, Maanshan 243000, China; 2 The State Key Laboratory of Mining Response and Disaster Prevention and Control in Deep Coal Mine, School of Earth and Environment, Anhui University of Science and Technology, Huainan 232001, China; 3 Collaborative Innovation Center of Recovery and Reconstruction of Degraded Ecosystem in Wanjiang Basin Co-Founded by Anhui Province and Ministry of Education, Anhui Normal University, Wuhu 241000, China; 4 Chuzhou Bureau of Ecology and Environment, Chuzhou 239000, China; 5 Institute of Environment-friendly Materials and Occupational Health, Anhui University of Science and Technology (Wuhu), Wuhu 241000, China; 6 Engineering Laboratory of Comprehensive Utilization and Ecological Protection of Soil and Water Resources in High Diving Level Mining Area of Anhui Province, Huainan 232001, China; 7 Key Laboratory of Bioresource and Environmental Biotechnology of Anhui Higher Education Institutes, School of Biological Engineering, Huainan Normal University, Huainan 232038, China; 8 Institute of Intelligent Machines, Chinese Academy of Sciences, Hefei 230031, China; 9 Department of Preventive Medicine, Bengbu Medical College, Bengbu 233033, China

**Keywords:** rice husk biochar, vermicompost, heavy metals, earthworm, industrial sludge

## Abstract

The aim of this study was to assess the total concentration and speciation variation of heavy metals (Pb, Cd, Cu and Zn) during composting and vermicomposting of industrial sludge with different addition rations of rice husk biochar. Results indicated that pH, EC, total phosphorus (TP) and total potassium (TK) were increased and total organic carbon (TOC) and total nitrogen (TN) were decreased during the composting of industrial sludge with biochar compared with the control (sludge without biochar). The addition of earthworm to the biochar-amended sludge further decreased pH and TOC but highly enhanced the EC, TN, TP and TK. Comparatively lower concentrations of total and DTPA-extractable heavy metals were observed in biochar-amended sludge treatments mixed with earthworm in comparison with the biochar-amended sludge treatments without earthworm or the control. Sequential extraction methods demonstrated that vermicomposting of sludge with biochar converted more metals bound with exchangeable, carbonate and organic matter into the residual fraction in comparison with those composting treatments of sludge with biochar. As a result, the combination of rice husk biochar and earthworm accelerated the passivation of heavy metals in industrial sludge during vermicomposting. Rice husk biochar and earthworm can play a positive role in sequestering the metals during the treatment of industrial sludge. This research proposed a potential method to dispose the heavy metals in industrial sludge to transform waste into resource utilization.

## 1. Introduction

Lots of industrial sludge is inevitably produced by various factories, such as tanning, fur finishing, coal manufacturing, electroplating and mechanical manufacturing processing [1]. One potential utilization of sludge is as the amendment to land for improving soil structure and fertility [2]. However, some heavy metals in sludge, such as Zn, Pb, Cu and Cd, can pose a serious risk to animal, plant and human health, hindering the utilization and disposal of the solid waste [3]. Therefore, it is necessary to seek effective strategies to decrease and remove these heavy metals from sludge [4].

In recent years, many technologies have been proposed to remove the heavy metals in sludge. Applying an amendment to the initial sludge is an effective way to reduce the metal content of the final product [5]. Phosphoric rock, fly ash, straw and sawdust are often used in the composting of sewage sludge. By changing and adjusting the physical and chemical properties via complexation, adsorption, iron exchange, precipitation and redox reactions, the mobility and toxicity of the metals decreased in sewage sludge [6,7]. Nowadays, biochar is recognized as a potential amendment by its large surface area, high stability, well-developed pore structure, high cation exchange capacity and large quantities of surface functional groups (e.g., phenolic, carbonyl, carboxylic and quinone groups). Previous works also reported that it could adjust the composting process of manure, agricultural wastes and sewage sludge by altering the physical and chemical properties of the substrate and decreasing the heavy metals’ bioavailability in the compost [8,9]. Steiner et al. [10] found that biochar made from organic materials can improve the physicochemical properties of poultry manure during composting. Agyarko-Mintah et al. [1] reported that co-composting raw poultry litter with biochar produced from green waste resulted in a high content of nutritional value of the compost product. Malińska et al. [11] observed that the application of biochar produced from willow woodchips to the composting of sewage sludge could adjust the heavy metal contents in the substrate.

Compared with traditional disposal methods, vermicomposting is a low-cost, eco-efficient organic waste treatment technology. It has received a lot of attention as an environmentally friendly method to dispose of sewage sludge. During vermicomposting, earthworms play a vital role in the substrate, as they can convert most of the organic matter into valuable products rich in phosphorus, nitrogen, potassium and humic substances [12]. The earthworm can not only absorb and accumulate the heavy metals from the substrate but also change the speciation of heavy metals in the final product [7]. Further, vermicomposting of sewage sludge with supplementary materials was proposed as a new way to change the availability of heavy metals in the substrate. The supplementary materials included crop straw, wood chips and saw dust, as well as soil and tea factory coal ash [13]. Malińska et al. [11] also reported that sewage sludge-derived biochar as a supplementary material vermicomposting with municipal sludge decreased the heavy metal mobility in the final product. Actually, the application of these supplementary materials in combination with the earthworm can further adjust the pH, OM and C/N ratio, and therefore eliminate the toxicity and change the mobility of heavy metals in the substrate [14]. 

Urban sludge can be divided into sewage sludge and industrial sludge. Although many previous studies have investigated the effects of biochar on the composting or vermicomposting of various organic wastes and sewage sludge, to our knowledge, no research has assessed the addition of biochar on the composting and vermicomposting of the industrial sludge and focused on the concentration and speciation of heavy metals in the industrial sludge during these processes. Therefore, the overall goal of this research was to present the results from the laboratory composting and vermicomposting of industrial biochar with sludge. The scope of this study included: (1) physical and chemical characteristics of the obtained compost and vermicompost of industrial sludge mixed with the biochar; (2) the effects of composting and vermicomposting with biochar on the distribution and bioavailability of Zn, Pb, Cd and Cu in the industrial sludge; and (3) the mechanisms of the combination of biochar and earthworm on the sequestration effects of heavy metals during vermicomposting of the industrial sludge.

## 2. Materials and Methods

### 2.1. Experiment Design

**Figure 1:**
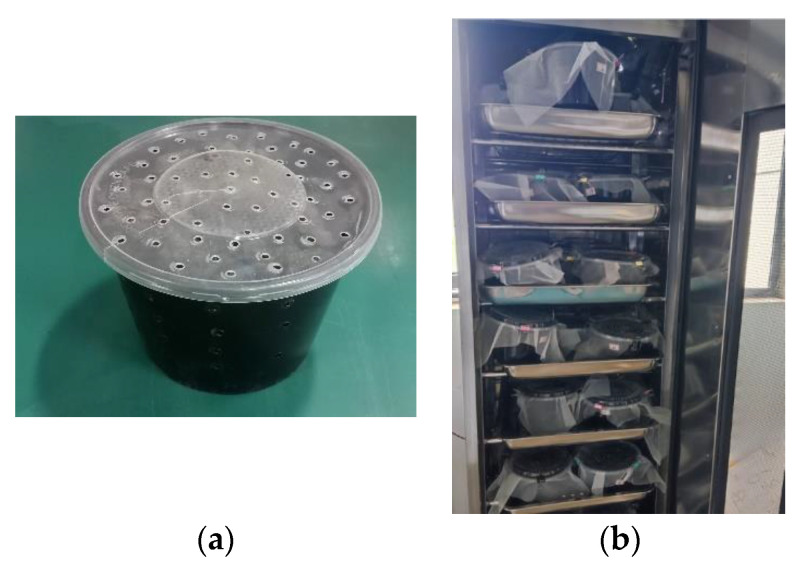
Composting devices: (**a**) culture container; (**b**) compost bin.

Compost and vermicompost treatments were performed in 0.5 L cylindrical culture containers with tight-fitting lids, drainage holes in the bottom and air vents on the lids and sides (Figure 1). Two-hundred-gram industrial mixtures for each treatment were filled in the containers. Three compost treatments (B1–B3) were prepared using the same biochar but with different proportions (2%, 4% and 8%). For vermicompost, according to the optimal stocking density of earthworm studied by Ndegwa et al. [15], 15 clitellated adult earthworms were added to the treatment (BE1–BE3) composted with different biochar additions. In order to prevent the earthworms from escaping, the containers were covered with fine gauze. All the containers were placed in the dark and kept at temperature 25 ± 3 °C and maintained at 65 ± 3% humidity. The composting treatments (B1–B3) and vermicomposting treatments (BE1–BE3) were incubated for 35 days, and all the treatments were replicated three times.

### 2.2. Substrate and Earthworm

The industrial sewage sludge was collected from a steel factory wastewater treatment plant in X city (Anhui Province, China). The rice hull biochar was produced in an experiment pyrolysis installation (at 600 °C). The rice hull biochar was crushed and sieved to pass through <2 mm to assume an even distribution in a mixture. The ration of biochar addition was established according to the studies reported by Malińska et al. [16] and He et al. [7]. The composition of materials in different treatments were listed in Table 1, where CK was set with no biochar and earthworm. Physicochemical properties of biochar and sludge are shown in Table 2.

In this study, *Eisenia fetida*, which showed high tolerance in sewage sludge in many other studies [17], was used for the vermicomposting of treatments (BE1–BE3). The earthworm *Eisenia fetida* was obtained from a cultivation farm in Jurong city in Jiangsu Province, China, with an average weight of 0.35 ± 0.05 g. The earthworm was cultured in the laboratory for a domestication procedure for 15 days before throwing it in the sludge.

### 2.3. Analytical Methods

At the end of composting, the sludge samples were collected from different treatments for analyzation. The pH and EC of the samples were analyzed by pH meter (PHS-2C, China) and conductivity meter (DDS-307, China), respectively. TOC content was determined by the loss of ignition with samples after treatment at 550 °C for 4 h. Total N, P and K were measured according to the method published by Page et al. [18]. The total heavy metals (Pb, Cd, Cu and Zn) were determined by the method of HF-HNO_3_-HClO_4_. The heavy metals (Pb, Cd, Cu and Zn) were also extracted using the diethylene-triaminepenta acetic acid (DTPA) extraction method described in Fernández-Gómez et al. [19]. The heavy metal fractions were conducted using the method proposed by Tessier et al. [20]. The exchangeable (F1), carbonate-bound (F2), iron–manganese oxide-bound (F3), organic-matter-bound (F4) and residual fractions (F5) in each scenario were sequentially extracted. The heavy metal (Pb, Cd, Cu and Zn) concentrations of all extracts were measured by ICP-OES (PerkinElmer, Optima 2100 DV). Limits of detection (LOD) of Cd, Pb, Cu and Zn were 0.002, 0.03, 0.002 and 0.002 ug mL^−1^, respectively.

### 2.4. Statistical Analysis

All statistical analyses were conducted in triplicate using the SPSS 19.0 software. One-way ANOVA following LSD test was used to test the differences in related parameters among different treatments. Findings were regarded as significant at *p* < 0.05.

## 3. Results and Discussion 

### 3.1. Physicochemical Properties of Industrial Sludge

**Figure 2:**
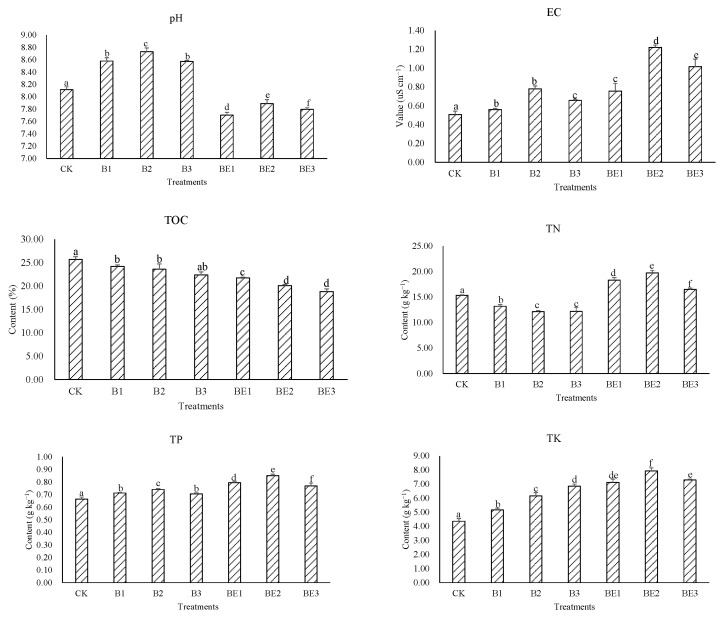
Physical and chemical properties of the sludge for different treatments. Data are presented as the mean ± SD for three duplicates. Columns with different letters show a significant difference at *p* < 0.05 level.

The basic physicochemical properties, such as pH, EC TOC, TN, TP and TK contents, are critical factors for heavy metal behaviors in the sludge during composting and vermicomposting. As shown in Figure 2, pH increased in B1–B3, and a peak value of pH was noted in B2 (8.58, *p* < 0.05), while it decreased from BE1–BE3 compared with CK (*p* < 0.05) and the lowest value of pH was observed in BE1 (7.70, *p* < 0.05), which suggests that application of rice husk biochar to sludge could increase the pH, whereas earthworm added in the biochar-amended sludge would decrease and readjust the pH. EC, TP and TK all increased from B1–B3 and BE1–BE3; both B2 and BE2 showed relatively higher values among B1–B3 (except TK in B3) and BE1–BE3 in comparison with CK (*p* < 0.05), respectively. Further, BE2 showed higher values of EC, TP and TK than those values in B2 (*p* < 0.05), denoting that the addition of 4% rice husk biochar increased the EC, TP and TK of the sludge, and 4% rice husk biochar combined with earthworm can synergistically highly increase these indices in the sludge. TOC decreased from B1–BE3 and minimum value was found in BE3 (18.83%, *p* < 0.05), which was 26.76% lower than in the CK (*p* < 0.05), suggesting that rice husk biochar addition could decrease the TOC in the sludge while biochar combined with earthworm would significantly increase the reduction effect. The TN in B1–B3 was significantly lower than in CK (*p* < 0.05) and the lowest level of TN was found in B2 (6.16 g kg^−1^, *p* < 0.05), but the TN showed the increasing values in BE1–BE3 with a maximum value of 19.76 mg kg^−1^ in BE2, which was 28.65% higher over the control (*p* < 0.05), suggesting that 4% rice husk biochar decreased the TN in the sludge, while rice husk biochar plus earthworm at the same addition ratio could promote the TN content during vermicomposting.

Because the rice husk biochar was alkaline (9.51 from Table 1), and the application of biochar can increase the retention of NH_4_^+^ to increase pH, high pH values were therefore found in B1–B3 during composting of sludge [10]. The decrease in pH in BE1–BE3 for the addition of earthworm into biochar-amended sludge could be due to organic matter in the sludge accelerated for mineralization by the synergistic effect from the earthworm, which released a lot of salt base ions and increased the pH of the sludge by ion exchange with hydrogen ions [21]. EC increased in treatments B1–BE6. Similar results were reported in previous works [7,14]. The increasing EC during the composting of biochar with sludge may be due to the fact that biochar accelerates the degradation of organic matter, leading to more mineral salts in available forms [14,22]. When adding the earthworm to the biochar-amended sludge, the activity of the earthworm could increase the activity and population number of the microorganisms, and also increase the metabolic release of low-molecular-weight organic acids and the generation of salt base ions, thus further increasing the EC content in the sludge [23]. In addition, earthworms can secrete calcium compounds [24], which could increase the EC of the sludge as well.

The TP increased from B1 to BE3 treatments as compared with CK. A similar trend was found in other studies [11,14,25]. During composting of biochar and sludge, TP concentration gradually increased in B1 to B3, which may be ascribed to the loss of C, H, O and dry mass as H_2_O, CO_2_, and H_2_S [26]. During vermicomposting of biochar and sludge from BE1 to BE3, the increase in TP concentration could be ascribed to the mineralization of OM [25]. In addition, as the earthworm was added to the biochar-amended sludge, some TP could also be released from the mobilization and mineralization of phosphorous by the earworm gut enzyme and microflora [14]. TK showed a similar trend to TP. The TK concentration in biochar (6.79 g kg^−1^) was higher than in sludge (4.85 g kg^−1^). Thus, the increase in TK concentration in B1–B3 may be due to the release of potassium from the rice husk biochar. Increasing TK concentrations in the compost and vermicompost mixtures could also be ascribed to the mineralization process [14]. Additionally, during vermicomposting, microbial and enzymatic activities in the earthworm gut would promote a higher rate of the mineralization process, which increased higher TK concentrations in the biochar-amended mixture [27].

TN decreased in B1–B3 during the composting of biochar and sludge. Similar results were also reported by Khan et al. [26]. The TN concentration in rice husk biochar (6.17 g mg^−1^) was lower than in sludge (19.10 g mg^−1^); therefore, biochar would present the dilution effect in the sludge. Godlewska et al. [28] found that some biochar is environmentally friendly to nitrifying bacteria and accelerates the process of denitrification. For the application of biochar to sludge, organic nitrogen was gradually converted into inorganic nitrogen, ammonium nitrogen shifted into ammonia, and nitrate nitrogen released N_2_O and other gases through denitrification, thus lowering the TN [7]. During the vermicomposting process, the TN values for BE1–BE3 were higher than the other treatments. The increase in TN could be due to nitrogen-containing mucus, growth hormones and enzymes secreted/excreted by the earthworms, and these nitrogen-containing substances can result in an increase in the nitrogen content in the sludge [29]. It is possible that in the presence of biochar, microorganisms in the earthworm gut are more abundant and nitrogen-processing enzymes are more active [26]. Similar results were also provided by Suthar [30], who described an increase in TN during the vermicomposting process.

In this study, the TOC contents decreased gradually during composting and vermicomposting of biochar with sludge, and earthworm activity resulted in a significant decrease in TOC in BE1–BE3 compared with other treatments. Similar results were supported by Zhang et al. [31]. The decrease in the organic matter and mineralization process by the indirect accelerating effect of biochar via the stimulation of microbial and enzymatic activity caused the reduction in TOC during the composting of sludge [32]. As the earthworm entered the biochar-amended sludge, TOC could also be lost as CO_2_ through the combined biological action of earthworms and microorganisms [7]. Gupta and Garg [33] stated that earthworms could create microclimatic conditions that favor microbial respiration and also promote TOC reduction.

Further, from the results of pH, TN, TP and TK during composting and vermicomposting, the addition rate of 4% biochar may create better conditions of sludge than an 8% dose, which denotes that an optimum dose of rice husk biochar at 4% would provide an appropriate compost and vermicompost environment for industrial sludge. Similar results were also reported by Paul et al. [25], who found that a little dose (5%) of biochar rather than a higher dose made from woody biomass of *Prosopis juliflora* improved the compost and vermicompost process better. In fact, different kinds of biochar contain macro- and micronutrients, which could favor the growth of microorganisms and macrofauna such as earthworms [9]. However, biochar contains some toxic compounds such as heavy metals, dioxins, polychlorinated biphenols and polycyclic aromatic hydrocarbons [16]. Elevated levels of biochar addition would also pose adverse effects to the microorganisms and earthworms. As a result, an appropriate dose is needed to achieve optimal usage of the biochar concerning compost and vermicompost. 

### 3.2. Heavy Metal Contents in Industrial Sludge

**Figure 3:**
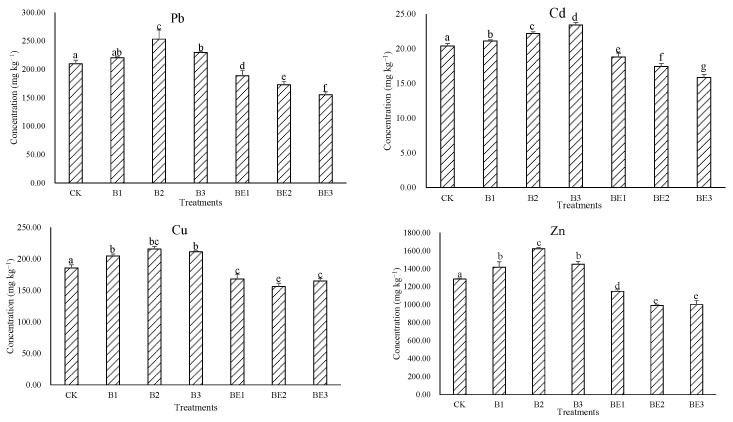
Total heavy metal concentrations in the sludge for different treatments. Data are presented as the mean ± SD for three duplicates. Columns with different letters show a significant difference at *p* < 0.05 level.

The total concentrations of heavy metals (Pb, Cu, Cd and Zn) in different treatments for compost and vermicompost of biochar and sludges were tested and are shown in Figure 3. Composting of biochar and sludge resulted in a typical increase in the concentrations of selected heavy metals compared with the control. The highest concentrations of Pb, Cu and Zn were detected in B2, whereas the highest Cd concentration was detected in B3 (*p* < 0.05). However, vermicomposting of biochar and sludge led to a reduction in the concentrations of the heavy metals in comparison with CK and the other treatments (*p* < 0.05). Minimum concentrations of Pb and Cd were noticed in BE3 (*p* < 0.05), while minimum concentrations of Cu (*p* < 0.05) and Zn were noticed in BE2, as compared with the control, respectively. 

In this study, biochar addition increased the total heavy metal concentrations in the compost of sludge. This could be due to the weight loss in organic matter resulting from the decomposition, release of CO_2_ and mineralization processes promoted by the application of biochar [14]. Furthermore, previous studies demonstrated that biochar displays a notable affinity to heavy metals [34]. During the composting of sludge amended with biochar, the metals would not disappear from the compost. It can, therefore, be supposed that an application of biochar during the composting of sludge would be conducive to the increment of heavy metals in the compost. Similar results were supported by Malinska et al. [16], who found that the addition of biochar made from willow woodchips increased the Cd, Cu, Zn, Mn, Cr and Pb concentrations during the composting of sewage sludge. During the process of the vermicomposting of sludge, the addition of biochar enhanced the secretion of the extra polymeric substance, which promoted the metabolism of the earthworm and accelerated the heavy metals that bioaccumulated in the tissues and gut/intestine of the earthworm [35]; thus, total heavy metal concentrations decreased in the biochar-amended sludge during vermicomposting. Similar results of reduction in total heavy metal concentrations for the vermicomposting of woody biochar-amended waste were also reported by Paul et al. [25].

Some countries have established limits of heavy metals for composted sludge. Compared with the Compost Quality Standards and Guidelines proposed by the New York State Association of Recyclers, USA (Pb: 300 mg kg^−1^, Cd: 39 mg kg^−1^, Cu: 1500 mg kg^−1^, Zn: 2800 mg kg^−1^) [36], the British Standard PAS 100 limits for Source Segregated Composted Waste (Pb: 200 mg kg^−1^, Cd: 1.5 mg kg^−1^, Cu: 200 mg kg^−1^, Zn: 400 mg kg^−1^) [37] and the Agricultural Sludge Pollutant Control Standard of China (Pb: 300 mg kg^−1^, Cd: 3 mg kg^−1^, Cu: 500 mg kg^−1^, Zn: 1200 mg kg^−1^) [38], the concentrations of Pb in the final compost and vermicompost of biochar-amended sludges did not exceed these limits. Although Cd concentrations in all treatments exceeded the Chinese standard and British Standard PAS 100 limits and were lower than USA limits, the vermicomposting treatments registered much lower Cd values. Cu concentrations in the earthworm-added treatments were lower than those three limits as compared with other treatments. Zn concentrations in vermicomposting treatments were much lower than the USA and China limits as well. Considering these guidelines, heavy metal concentrations in the industrial sludge were significantly decreased by the addition of biochar and earthworm, and the sludge needed to be treated for a longer period to produce a lower-metal-concentration environment before it could be safely used in other aspects, such as potting media in horticulture or as an organic fertilizer in agriculture fields.

### 3.3. Bioavailability of Heavy Metals in Industrial Sludge 

**Figure 4:**
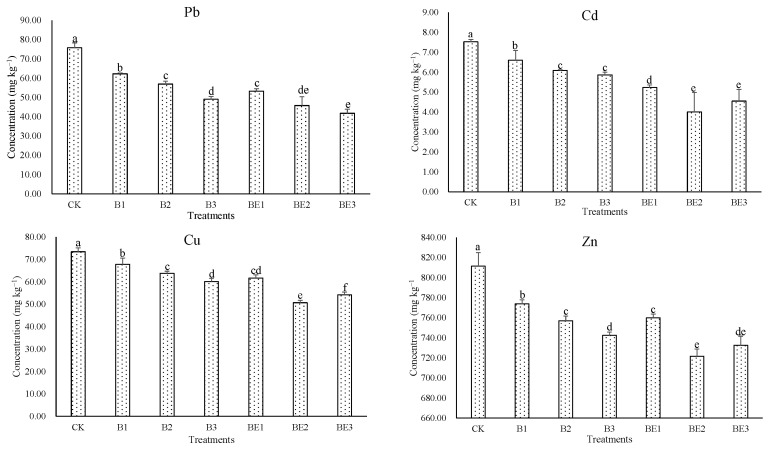
DTPA-extractable heavy metal concentrations in sludge for different treatments. Data are presented as the mean ± SD for three duplicates. Columns with different letters show a significant difference at *p* < 0.05 level.

The bioavailability of heavy metals is considered as the special fraction of heavy metals easily taken up by the organisms. It provides a better indicator of metal toxicity than total metal concentrations. The bioavailability of heavy metals during the composting and vermicomposting of the sludge depends on the type of amendment [23]. The DTPA-extractable method is always adopted to assess the bioavailable heavy metals in the substrate because the total heavy metal concentrations increased during the composting or vermicomposting, ascribing to the loss of weight and volume of the final biosolid [39]. The changes of bioavailable heavy metal concentrations in different treatments were shown in Figure 4. The DTPA-extractable heavy metal contents in all biochar-amended treatments were significantly decreased compared with the control (*p* < 0.05). The minimum DTPA-extractable heavy metal concentrations were recorded in B3 for composting of biochar and sludge. Lowest values of DTPA-extractable heavy metals were noticed in BE2 for vermicomposting of biochar and sludge (except DTPA-extractable Pb in BE3). Actually, DTPA-extractable heavy metal concentrations in vermicomposting treatments were significantly lower than those in composting treatments (*p* < 0.05, except DTPA-extractable Zn in B3 and BE3), which suggested that biochar was effective to decrease the bioavailability of heavy metals in industrial sludge during composting, and biochar and earthworm could synergistically further reduce the bioavailable heavy metals in the industrial sludge during vermicomposting. Our findings were similar with other researchers [8,26,40], who observed the influence of biochar made from wheat straw on the bioavailability reduction for heavy metals in the dewatered fresh sewage sludge during composting, and that bamboo biochar plus *Eisenia fetida* synergistically decreased the bioavailability of heavy metals in green waste during vermicomposting.

The correlations between DTPA-extractable heavy metals and chemical indices of sludge during composting and vermicomposting are shown in Table 3. TOC and TK were significantly positively correlated with DTPA-extractable heavy metals (*p* < 0.05 or *p* < 0.01), TN was significantly negatively correlated with DTPA-extractable Cd and Zn (*p* < 0.05) during composting of biochar and sludge, whereas in vermicomposting treatments, pH, EC, TOC and TK all showed significant correlations with DTPA-extractable heavy metals (*p* < 0.05 or *p* < 0.01, except DTPA-extractable Pb with EC and TK). Therefore, the addition of biochar to industrial sludge during composting and vermicomposting presented two different ways in immobilizing the heavy metals in the sludge. 

Actually, the organic matter of biochar contains many functional groups, such as carboxyl groups and hydroxyl groups, which can bind metal ions to form organic–metal complexes. Therefore, the TOC of biochar affects the heavy metal bioavailability in the sludge [41]. The biochar can also alter cation exchange between metal ions and mineral ions (e.g., K^+^) that influence the metal mobility [42]. The passivation of heavy metals after application of biochar to composted sludge can also occur through the formation of complexes, precipitation on biochar surface, physical sorption, electrostatic interactions with the charge on biochar surface and the increasing pH [34,43]. Previous works also approved that biochar addition during composting of sewage sludge and animal breeding led to a reduction in the mobility of Pb and Ni [40] and Cu and Zn as well [44,45]. When earthworm was added to the substrate of biochar and sludge, the passivation of heavy metals was strengthened and can be postulated that earthworm could excrete metallothionein and mucus, which could bind the heavy metals in the substrate and further alter the pH, EC, TOC and TK of the substrate, which results in the reduction in the availability of heavy metals in the sludge [23,46,47]. Some heavy metals can be absorbed by the earthworm and bound by the small protein rich in cysteine, such as metallothionein, in the tissues of the earthworm [7]. Similar results were also reported by Zhang et al. [31] that earthworm changed the TOC, EC and pH, inhibiting the bioavailability of Zn and Cd during the vermicomposting of pure sludge.

### 3.4. Heavy Metal Speciation in Industrial Sludge

Sequential extraction of heavy metals in sludge is usually adopted to assess the metal fractions in sewage sludge [23]. In this research, sequential extraction was used to evaluate whether earthworm and biochar could synergistically affect the fraction distribution of heavy metals in industrial sludge. Both the composting and vermicomposting treatments were subjected to the sequential extraction, in which metals were classified as exchangeable (F1), bound to carbonates (F2), bound to iron–manganese oxides (F3), bound to organic matter (F4) and residual (F5).

**Figure 5:**
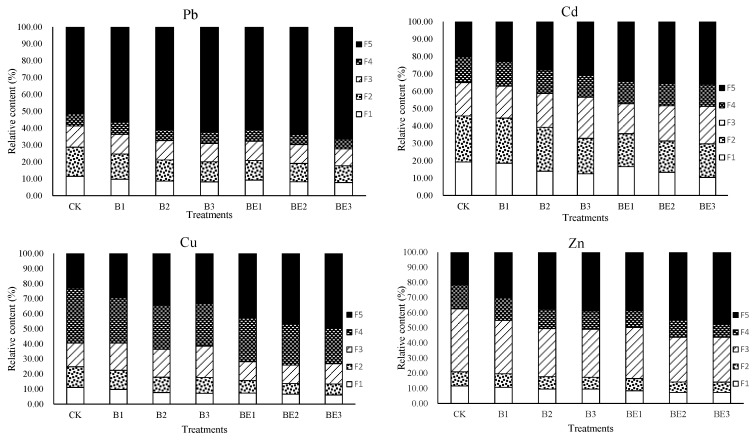
Fractionation of heavy metals in industrial sludge for different treatments. Heavy metal fractions are F1 (exchangeable), F2 (carbonate-bound), F3 (Fe–Mn oxides), F4 (organically bound) and F5 (residual).

The sequential extraction results for Pb, Cr, Cu and Zn in the industrial sludge are shown in Figure 5. The distributions of heavy metals in each fraction of industrial sludge varied among different treatments. Pb in the F1, F2, F3 and F4 fractions decreased, while Pb in the F5 fraction increased with increasing biochar ratio in composting and vermicomposting treatments compared with the control. Additionally, Pb in the F1, F2, F3 and F4 fractions of vermicomposting treatments (BE1–BE3) decreased less compared with the corresponding composting treatments (B1–B3), respectively. Similarly, Pb in the F5 fraction increased more in vermicomposting treatments (BE1–BE3) than in those of composting treatments (B1–B3). Cd in fractions F1, F2 and F4 decreased in B1–B3 and decreased further in BE1–BE3, while Cd in fraction F5 increased in B1–B3 and increased further in BE1–BE3. Cd in the F3 fraction was slightly varied among different treatments.

Cu in the F1, F2 and F4 fractions decreased during the composting of biochar and sludge (B1–B3) compared with CK, followed by a further decrease during the vermicomposting of biochar and sludge (BE1–BE3). However, Cu in the F5 fraction changed in the reverse direction in comparison with Cu in the F1, F2 and F4 fractions. Cu in the F3 fraction in B1–B3 increased and those proportions were higher than CK, but were followed by a decrease in BE1–BE3, and those proportions were lower than CK. Zn in fractions F1, F2, F3 and F4 in B1–B3 decreased and were lower than CK, followed by a further decrease in BE1–BE3, while Zn in the F5 fraction increased in B1–B3 and further increased in BE1–BE3, which were all higher than CK.

Ascribing to the variation of heavy metal fractions in the biochar-amended sludge during composting and vermicomposting, the mobility factors (MFs) of heavy metals proposed by Narwal et al. [48] are calculated in Table 4. Applying 2–8% biochar to the sludge decreased the MFs of Pb, Cd, Cu and Zn by 14.08–30.19%, 2.67–28.17%, 9.61–28.62% and 6.22–17.55%, respectively, compared with the control during composting. When biochar and earthworm were added into the sludge, the MFs of Pb, Cd, Cu and Zn were further decreased, and the addition of 8% biochar in combination with earthworm reduced the MFs of Pb, Cd, Cu and Zn in the sludge by 38.36%, 35.06%, 46.37% and 32.68% during vermicomposting, respectively, in comparison with the control (*p* < 0.05). Therefore, biochar could passivate the heavy metals in the sludge during composting, and biochar and earthworm could significantly and synergistically immobilize the heavy metals in the sludge during vermicomposting. 

Recently, lots of studies have reported that biochar is effective in passivating heavy metals, thus immobilizing the bioavailability of heavy metals in the substrate [22,28]. Biochar has many properties conducive to the immobilization of heavy metals. For example, the high levels of nitrogen and extractable inorganic nutrients such as potassium, phosphorus, magnesium and calcium in biochar, which are mostly alkaline, will promote the pH and immobilize the heavy metal activation [42]. A large amount of oxygen-containing functional groups on the surface of biochar also resulted in the immobilization of heavy metals [23,49]. Thus, biochar immobilizes heavy metals by converting the available fractions to geochemically more stable residual fractions, and therefore decreases their bioavailability and mobility [43]. Karimi et al. [50] found that increasing rates of biochar application could significantly decrease the concentrations of Pb and Cd in the exchangeable and carbonate forms but increase the residual forms. Park et al. [51] observed that chicken manure biochar was effective at decreasing the extractable concentrations of Pb, Cu and Cd. Jin et al. [52] discovered that chicken litter biochar changed the extractable Cd into the residual fraction. Jiang et al. [49] found that soluble Cu and Pb significantly decreased, while the reducible and oxidizable Cu and Pb increased due to biochar application. In this study, similar results showed that applying biochar to the sludge during composting decreased the heavy metals bound with exchangeable and carbonate fraction, but increased the heavy metals associated with the residual fraction. Meanwhile, biochar addition decreased the heavy metals bound with the organic fraction in this research, which was not in agreement with other studies that organic matter fractions were increased after the addition of biochar [50,52]. It was postulated that the addition of biochar to the industrial sludge could cause an indirect accelerating effect on the organic matter degradation via stimulating the microbial and enzymatic activity, which in turn caused the decreasing concentration of heavy metals bound to organic matter [44,53].

Straw, phosphoric rock, fly ash and sawdust were applied to the vermicompost to adjust the bioavailability of heavy metals in sewage sludge [7,23]. The additive materials and earthworm always showed synergistic effects on the transformation of heavy metal forms in the sludge [28]. In this study, adding the earthworm to the biochar-amended industrial sludge further decreased the F1, F2 and F4 and further increased the F5, suggesting that earthworm strengthened the passivation effects of heavy metals in biochar-amended sludge. The mechanism for the conversion of heavy metal forms during vermicomposting could be multifold, with the impacts from pH, EC, TOC and inorganic matters, such as TK, etc., as shown in Figure 2. Moreover, the decrease in heavy metals bound with extractable and carbonate fractions could be due to the bioaccumulation (therefore sequestration) of earthworms within their tissue via skin/gut absorption [54]. Heavy metal speciation can be affected by binding in the chloragogenous tissue of earthworms as well [55]. The earthworm activity affects the microbial activity and microbial population, which would incentivize the decomposition of the TOC [56]; meanwhile, with the humification role of earthworm during vermicomposting, organo–metal complexes with metal elements were changed; thus, heavy metal associated with organic matter varied [57]. Earthworms can also affect the pH in the vermicompost, which change the metal mobility in the substrate due to the impact of metal speciation via competitive complexation and sorption, etc. [41,58]. The increased residual fraction could be ascribed to the earthworm stimulating microbial activity, thus enzymatically degrading organic matter in the vermicompost bulk, which releases metal ions into the substrate [46]. After then, the released metal ion tends to be associated with humus, and the concentration of these metal-bound substances formed in the residual form increases during vermicomposting [39]. He et al. [7] also reported the same phenomenon that the residual fraction of nine heavy metals (As, Cr, Cd, Cu, Fe, Mn, Ni, Pb and Zn) in all trials were found to be increased by the addition of additive materials during vermicomposting.

## 4. Conclusions

The application of biochar to industrial sludge increased pH, EC, TP and TK but reduced TOC and TN, while applying the biochar and earthworm to the sludge further decreased pH and TOC but highly enhanced EC, TN, TP and TK. The addition of biochar increased the total metals but decreased the DTPA-extractable heavy metals in the sludge, while the application of biochar and earthworm decreased the total heavy metals and further reduced the DTPA-extractable heavy metals in the sludge. The metals bound with extractable, carbonate and organic fractions were decreased and with the residual fraction were increased by the addition of biochar. However, biochar combined with earthworm showed synergetic effects on the metal speciation in the sludge, which further decreased the metals associated with the exchangeable, carbonate and organic fractions and further increased the residual fraction. Therefore, the vermicomposting of biochar and sludge can effectively remove the heavy metals and alleviate the bioavailability of heavy metals in industrial sludge.

## Figures and Tables

**Table 1:** Composition of different treatments used in this study.

Sample	Composition		
	Industrial Sewage Sludge (g)	Rice Hull Biochar (g)	Earthworm (num)
CK	200	0	0
B1	196	4	0
B2	192	8	0
B3	184	16	0
BE1	196	4	15
BE2	192	8	15
BE3	184	16	15

**Table 2:** Physicochemical properties of industrial biochar and sludge used for different treatments.

Parameters	Industrial Sewage Sludge	Rice Hull Biochar
pH	7.7 ± 0.01 ^a^	9.51 ± 0.09
Moisture content (%)	78.24 ± 0.71	7.82 ± 0.28
EC (ms cm^−1^)	0.45 ± 0.01	0.32 ± 0.01
TOC (%)	28.76 ± 0.70	38.39 ± 0.60
TN (g kg^−1^)	19.10 ± 0.49	6.17 ± 0.46
TP (g kg^−1^)	0.75 ± 0.04	0.07 ± 0.01
TK (g kg^−1^)	4.85 ± 0.41	6.79 ± 0.5
Pb_total_ (mg kg^−1^)	189.73 ± 1.08	0.25 ± 0.01
Cd_total_ (mg kg^−1^)	19.83 ± 0.47	0.02 ± 0.01
Cu_total_ (mg kg^−1^)	164.05 ± 0.77	0.17 ± 0.02
Zn_total_ (mg kg^−1^)	1242.56 ± 3.87	0.15 ± 0.02

^a^ Values are mean ± standard deviation based on 3 samples.

**Table 3:** Pearson correlation between DTPA-extractable heavy metals and the physicochemical indices of sludge during composting and vermicomposting.

Item	Bioavailable Metal	pH	EC	TOC	TN	TP	TK
Compost	DTPA-extractable Pb	0.113	−0.32	0.856 **	0.615	0.254	−0.961 **
	DTPA-extractable Cd	−0.223	−0.647	0.725 *	0.757 *	−0.09	−0.972 **
	DTPA-extractable Cu	0.023	−0.443	0.685 *	0.644	0.097	−0.871 **
	DTPA-extractable Zn	−0.032	−0.433	0.725 *	0.724 *	0.15	−0.917 **
Vermicompost	DTPA-extractable Pb	−0.672 *	−0.611	0.872 **	0.288	0.071	−0.331
	DTPA-extractable Cd	−0.704 *	−0.833 **	0.692 *	−0.139	−0.304	−0.679 *
	DTPA-extractable Cu	−0.849 **	−0.914 **	0.694 *	−0.23	−0.458	−0.818 **
	DTPA-extractable Zn	−0.876 **	−0.958 **	0.729 *	−0.258	−0.498	−0.765 *

** means correlation is significant at the 0.01 level, * means correlation is significant at the 0.05 level.

**Table 4:** Mobility factors of heavy metals in industrial sludge for different treatments.

Treatments	Mobility Factors			
	Pb	Cd	Cu	Zn
CK	28.72 ± 1.43 a^†^	45.75 ± 2.28 a^††^	24.80 ± 1.23 a	20.94 ± 1.04 a
B1	24.68 ± 1.23 b	44.52 ± 2.21 a	22.41 ± 1.11 b	19.64 ± 0.98 a
B2	21.16 ± 1.05 c	39.22 ± 1.96 b	17.82 ± 0.88 c	17.75 ± 0.88 b
B3	20.05 ± 0.99 cd	32.86 ± 1.30 cd	17.70 ± 0.88 c	17.27 ± 0.86 b
BE1	20.91 ± 1.04 c	35.62 ± 1.78 c	15.68 ± 0.78 d	16.49 ± 0.82 b
BE2	19.11 ± 0.95 cd	31.37 ± 1.56 d	13.65 ± 0.68 e	14.19 ± 0.71 c
BE3	17.71 ± 0.88 d	29.71 ± 1.48 d	13.30 ± 0.66 e	14.10 ± 0.71 c

MF (mobility factor) = (F1 + F2)/(F1 + F2 + F3 + F4 + F5) × 100. ^†^ mean ± SD values (*n* = 3). ^††^ values followed by different letters differ significantly with *p* < 0.05.

## Data Availability

All data generated or analyzed during this study are included in this article.
